# Racial Disparities in Infliximab Efficacy for Ulcerative Colitis: Evidence Synthesis and Effect Modification Assessment

**DOI:** 10.3390/jcm13020319

**Published:** 2024-01-05

**Authors:** Stefanos Bonovas, Andreas G. Tsantes, Rozeta Sokou, Argirios E. Tsantes, Georgios K. Nikolopoulos, Daniele Piovani

**Affiliations:** 1 Department of Biomedical Sciences, Humanitas University, Pieve Emanuele, 20072 Milan, Italy; daniele.piovani@hunimed.eu; 2 IRCCS Humanitas Research Hospital, Via Manzoni 56, Rozzano, 20089 Milan, Italy; 3 Microbiology Department, “Saint Savvas” Oncology Hospital, 11522 Athens, Greece; andreas.tsantes@yahoo.com; 4 Neonatal Intensive Care Unit, “Agios Panteleimon” General Hospital of Nikea, 18454 Piraeus, Greece; sokourozeta@yahoo.gr; 5 Laboratory of Haematology and Blood Bank Unit, “Attiko” Hospital, School of Medicine, National and Kapodistrian University of Athens, 12462 Athens, Greece; atsantes@yahoo.com; 6 Medical School, University of Cyprus, Nicosia 2029, Cyprus; nikolopoulos.georgios@ucy.ac.cy

**Keywords:** inflammatory bowel disease, ulcerative colitis, race, infliximab, biologics, interaction, effect modification

## Abstract

An increasing amount of research explores the role of race in clinical phenotypes and outcomes in ulcerative colitis (UC). We aimed to investigate racial differences in infliximab (IFX) treatment efficacy in UC. We used aggregate data from IFX trials and evidence synthesis methods to generate race-specific efficacy estimates. Then, we tested the effect modification by race by comparing the race-specific estimates derived from independent evidence syntheses. We computed ratios of relative risks (RRRs) and performed tests of statistical interaction. We analyzed data from five randomized, placebo-controlled trials evaluating IFX as induction and maintenance therapy for adults with moderate-to-severe UC (875 participants; 45% Asians). We found no substantial evidence of racial differences concerning the efficacy of IFX in inducing clinical response (RRR = 0.89, 95% CI: 0.66–1.20; *p* = 0.44), clinical remission (RRR = 0.58, 95% CI: 0.24–1.44; *p* = 0.24), and mucosal healing (RRR = 0.99, 95% CI: 0.69–1.41; *p* = 0.95), or maintaining clinical remission (RRR = 0.81, 95% CI: 0.46–1.42; *p* = 0.45) and mucosal healing (RRR = 0.84, 95% CI: 0.48–1.46; *p* = 0.53), between Asian and Caucasian populations. Future clinical studies should expand the participation of racial minorities to comprehensively assess potential racial differences in the effectiveness of advanced therapies, including IFX, in the context of treating UC.

## 1. Introduction

Ulcerative colitis (UC) is a chronic inflammatory bowel disease affecting the colon and rectum to varying extents and leading to long-term complications and disability [1]. It follows a remitting and relapsing course. Symptoms usually develop over time and often include diarrhea, fever, urgency, tenesmus, abdominal cramps, rectal bleeding, weight loss, and fatigue, which vary in severity based on the extent of inflammation [1,2]. UC’s incidence and prevalence are rising globally, posing a major health challenge [3,4]. Its pathogenesis remains unclear; however, mucosal immune dysregulation, gut microbiota, genetic predisposition, and environmental exposures have been strongly implicated [1,5,6,7]. Its diagnosis relies on clinical symptoms and biologic, endoscopic, and histological findings; however, there is no gold standard [1,2]. UC imposes a substantial global economic burden and severely impacts the quality of life for those affected [8,9].

Treatment decisions, considering factors like disease activity, extent, progression, and patient preferences, play a pivotal role [10,11,12]. While effective for many, a significant proportion of UC patients fail to improve, leading to the emergence of advanced therapies like infliximab (IFX), golimumab, adalimumab, vedolizumab, ustekinumab, tofacitinib and others. These biologic and small-molecule drugs target various points in the inflammatory cascade and constitute the product of our enhanced comprehension of the mechanisms underlying UC. They enabled better control of the disease, leading to increased rates of clinical response and remission, endoscopic healing, and corticosteroid-free remission and, ultimately, an improvement in quality of life [9,13,14]. However, there remains uncertainty regarding the efficacy of many of these therapies in certain patient groups and disease phenotypes.

Among these therapies, IFX was the first biologic to receive United States Food and Drug Administration (FDA; September 2005) and European Medicines Agency (EMA; March 2006) approvals for the treatment of adults with moderately to severely active UC who had an inadequate response to conventional therapy. It is a purified recombinant DNA-derived chimeric IgG monoclonal antibody, with murine and human components, acting to inhibit tumor necrosis factor-alpha (TNF-a) [15,16]. IFX is very useful for neutralizing TNF and inhibiting immune responses in UC [17]. It has demonstrated efficacy and safety [18] and is widely used for treating UC patients, both for induction and maintenance of remission.

In recent years, there has been an expanding body of research on the role that race and ethnicity might play in affecting clinical phenotypes and outcomes in UC. Several studies comparing disease extent revealed distinctive patterns among racial groups. Black patients exhibited higher rates of proctitis and left-sided colitis compared to White patients, who were more likely to suffer extensive colitis [19,20,21]. Hispanic patients showed higher rates of extensive colitis compared with White patients [22]; however, other studies found no differences in disease location [23,24,25]. Asian populations with UC were found to have extensive colitis less frequently than other races [26], while those from South Asia were found to have significantly more pancolitis and less proctitis compared with those from East Asia [27]. On the other hand, examination of disease outcomes revealed interesting findings too: Extraintestinal manifestations, including arthritis and primary sclerosing cholangitis, were more common in Black than among White patients with UC [28]. Hispanic patients showed higher rates of colectomy compared with White patients [22], whereas Asians exhibited a lower likelihood of undergoing surgery for UC compared with Caucasians [27]. This review addresses a crucial research gap, exploring the influence of race on medication efficacy in UC and determining optimal medications based on race. While acknowledging the limitations of using race as a proxy for biological differences, it is important to note that in various diseases, race has proven to be a valuable marker for predicting the response to drug therapy [29].

Recently, Greywoode and colleagues performed the first study assessing the influence of race on the response to biologics [30]. Specifically, they conducted a post hoc analysis of randomized clinical trials in UC to assess the impact of race on response to golimumab. Their study showed that patients from racial minorities, primarily Asians, were less likely to achieve and maintain clinical response, clinical remission, and endoscopic healing when treated with golimumab compared with White/Caucasian patients. However, their analysis did not extend to IFX due to the limited representation of participants from racial and ethnic minority groups in their dataset who had received this treatment.

To address this gap, we utilized aggregate data reported from randomized, placebo-controlled IFX trials and evidence synthesis methodology to generate race-specific efficacy estimates and examine the effect modification by race (i.e., the interaction between treatment and race) to assess the potential influence of race on the efficacy of IFX therapy in UC.

## 2. Materials and Methods

### 2.1. Data Sources

To investigate potential racial differences in the efficacy of IFX as induction and maintenance therapy in UC, we used data from randomized, placebo-controlled studies identified through a technical review published recently by our research group [31]. The search was extended up to October 2023, including PubMed, Embase, Scopus, and ClinicalTrials.gov, with search algorithms combining *infliximab* and *ulcerative colitis* (limited to randomized clinical trials), yet it yielded no additional IFX studies.

Data were independently extracted in duplicate (A.G.T. and R.S.) and recorded in a Microsoft Excel sheet. This included information such as citation data, trial acronym, first author’s surname, year of publication, geographical setting, duration of follow-up, number of patients, population characteristics, ethnicity/race details, and intervention specifics, including the IFX’s dosage and administration. We exclusively considered efficacy data relating to dosage and administration as specified in the Summary of Product Characteristics (SPC), specifically intravenous infusions of IFX 5 mg/kg at weeks 0, 2, and 6 (for induction) and every 8 weeks thereafter (for maintenance of remission).

### 2.2. Quality Assessment

Two authors (A.G.T. and R.S.) independently assessed the risk of bias (RoB) for each trial (induction and maintenance phase, separately) using the Cochrane RoB tool [32]. This assessment covered six key domains: sequence generation, allocation concealment, blinding, incomplete outcome data, selective outcome reporting, and other potential sources of bias. Each domain was scrutinized and classified as adequate (low RoB), inadequate (high RoB), or unclear (uncertain RoB). Disagreements in data extraction, or in RoB assessment, were resolved via discussion between the authors, aiming to reach a consensus. If needed, a third author (S.B.) was consulted to facilitate resolution.

### 2.3. Racial Categories

Race was determined based on information provided in the clinical trials’ published reports. When the vast majority of individuals (>90%) belonged to a specific race, the entire population was categorized as belonging to that particular race. In this analysis, race was categorized as Asian (including Chinese and Japanese patients) vs. Caucasian (encompassing patients from Europe, America, Oceania, and the Middle East). It was not possible to analyze race-specific efficacy for Black and other racial groups.

### 2.4. Outcomes Assessed

We assessed the following efficacy outcomes: (i) clinical response, clinical remission, and mucosal healing at the end of the induction phase (week 8), and (ii) clinical remission and mucosal healing at the completion of the maintenance phase. Clinical response is defined as a reduction from the baseline in the Mayo Clinic score [33] by a minimum of 3 points and at least 30 percent, along with a reduction in the rectal bleeding subscore of at least 1 point or achieving an absolute rectal bleeding subscore of 0 or 1 point. Clinical remission is defined as a Mayo Clinic score of 2 points or lower, with no individual subscore exceeding 1 point. Mucosal healing is defined as achieving an absolute endoscopy subscore of 0 or 1 point.

### 2.5. Statistical Methods

The risk ratio (RR) was the metric used to quantify treatment effects. RRs with 95% confidence intervals (CIs) were computed adhering to the intention-to-treat principle. Random-effect models were employed to synthesize evidence. Race-specific pooled RRs were calculated using the Mantel–Haenszel method [34], and estimates of heterogeneity were determined with the restricted maximum likelihood approach [35].

Inconsistency among the trial results was assessed using the I-squared statistic [36]. We interpreted heterogeneity, based on the I-squared, as follows: 0–40%, unimportant heterogeneity; 30–60%, moderate; 50–90%, substantial; and 75–100%, considerable heterogeneity. Due to the limited number of studies included in each evidence synthesis, it was not possible to perform a formal assessment of small-study effects. Generally, tests for funnel plot asymmetry are applied when there are a minimum of ten studies included in the analysis. Otherwise, these tests lack the statistical power to reliably distinguish real asymmetry from chance [37].

To examine for potential racial differences in treatment efficacy of IFX between Asians and Caucasians, we compared the race-specific RRs, derived from independent evidence syntheses, with a test of interaction [38]. We reported the ratio of race-specific RRs, *z*-score, and respective *p*-value for each one of these comparisons. A ratio of RRs equal to 1.00 indicates that the estimated treatment effect size is the same in Asians and Caucasians; the further the ratio of RRs is from 1.00, the greater the influence of race on the treatment’s efficacy in UC is. When the ratio of the RRs was >1.00, it indicated a better treatment effect in Asian populations; when it was <1.00, it showed a better treatment effect in Caucasian patients.

We used R statistical software [39], version 4.0.3, for conducting our analysis. All *p*-values were 2-tailed, and in all tests, a *p*-value < 0.05 signified statistical significance.

## 3. Results

We extracted and utilized aggregate data from four publications [40,41,42,43] reporting the results of five multicenter, randomized, placebo-controlled trials (Jiang et al. [40], Xi’an Janssen [41], Kobayashi et al. [42], ACT 1 [43], and ACT 2 [43]; Table 1) that assessed IFX as induction and maintenance therapy for adults with moderate-to-severe UC (i.e., Mayo score of 6 to 12 points, with an endoscopic subscore of at least 2 points) who had not been previously exposed to biologics. Permitted concomitant medications included corticosteroids, aminosalicylates, and immunosuppressants. Their doses remained constant except for corticosteroids, which were tapered. We analyzed data on 875 patients (Asians, 44.5%).

In these five trials, the mean age of participants spanned a range of 34 to 42 years, while the mean disease duration varied from 4 to 8 years. The percentage of female patients fluctuated between 36% and 40%, and the follow-up ranged from 26 to 54 weeks. Three of the studies [40,41,42] exclusively recruited Asian populations, specifically Chinese [40,41] and Japanese patients [42], while the remaining two studies (ACT 1, ACT 2 [43]) enrolled patients from Argentina, Australia, Austria, Belgium, Canada, Czech Republic, Denmark, England, France, Germany, Israel, Italy, the Netherlands, New Zealand, Scotland, Spain, Switzerland, and the United States, the vast majority of whom (>90%) were Caucasians. The studies exhibited homogeneity in terms of outcomes’ definitions. All studies contributed to all five efficacy outcomes.

### 3.1. Quality Assessment

**Figure 1:**
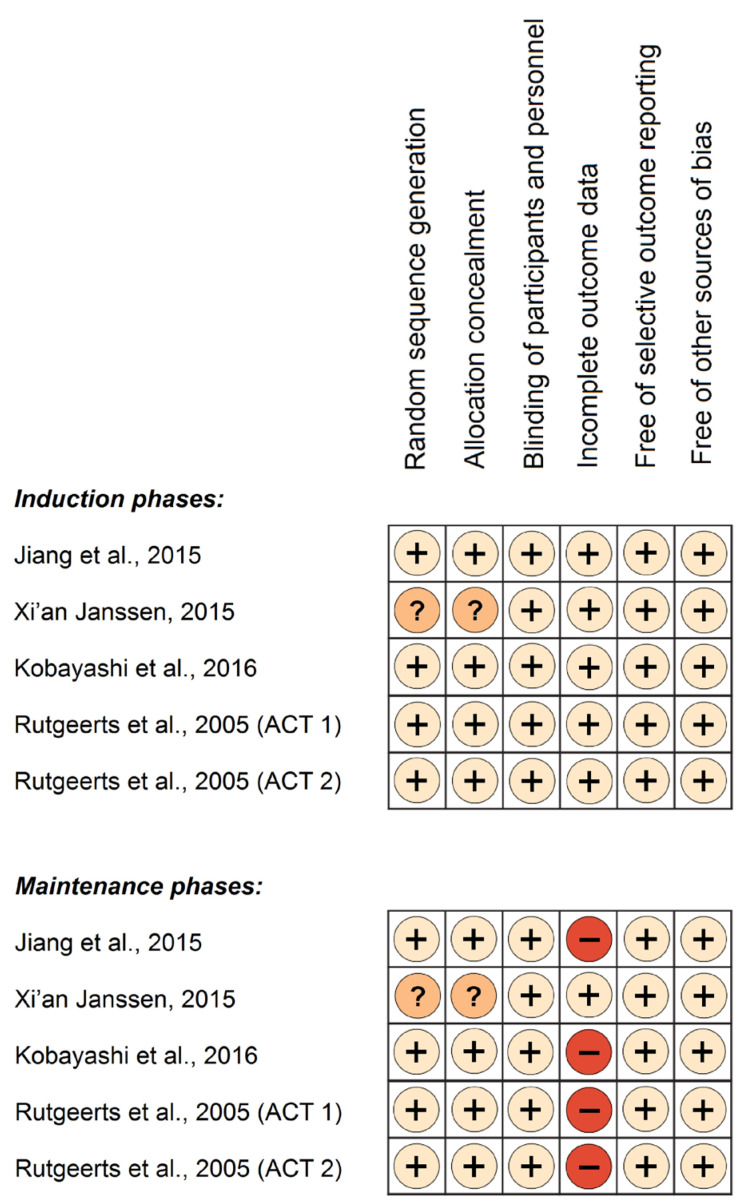
Risk of bias assessment of clinical trials’ induction and maintenance phases. Symbols: (+), low risk of bias; (−), high risk of bias; (?), unclear risk of bias, [40,41,42,43].

RoB assessment of the studies revealed low risk across the induction phases, but high risk across the maintenance phases with regard to the “incomplete outcome data” domain. Though follow-up rates were consistently high in the induction phases and equally balanced between study groups, there was a notable decline in complete follow-up during the maintenance phases. This decline was accompanied by significant imbalances across study groups, and unequal drop-out rates attributed to adverse effects. These disparities in study withdrawals between groups raise the possibility for biased effect estimates. The RoB assessment is presented in Figure 1, for both the induction and maintenance phases.

### 3.2. Assessment of Racial Differences in Treatment Efficacy of IFX

#### 3.2.1. Induction of Clinical Response

**Figure 2:**
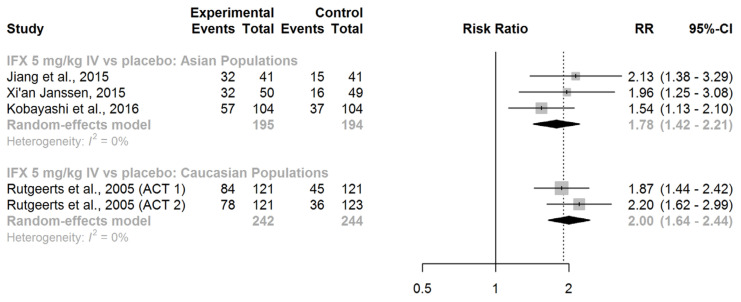
Induction of clinical response. Abbreviations: IFX, infliximab; IV, intravenous; RR, risk ratio; CI, confidence interval, [40,41,42,43].

IFX (given intravenously at a dose of 5 mg/kg at weeks 0, 2, and 6) showed significant superiority over placebo in inducing clinical response in adult patients with moderately to severely active UC who had not previously received any biologic therapy. This efficacy was observed in both Asian (RR = 1.78, 95% CI: 1.42–2.21) and Caucasian populations (RR = 2.00, 95% CI: 1.64–2.44), as shown in Figure 2.

A comparison of the race-specific pooled effect estimates did not reveal any difference (ratio of RRs = 0.89, 95% CI: 0.66–1.20; z-score = −0.77; *p*-value for interaction = 0.44). Thus, we found no evidence of effect modification by race concerning the efficacy of IFX in inducing clinical response in patients with active UC.

#### 3.2.2. Induction of Clinical Remission

**Figure 3:**
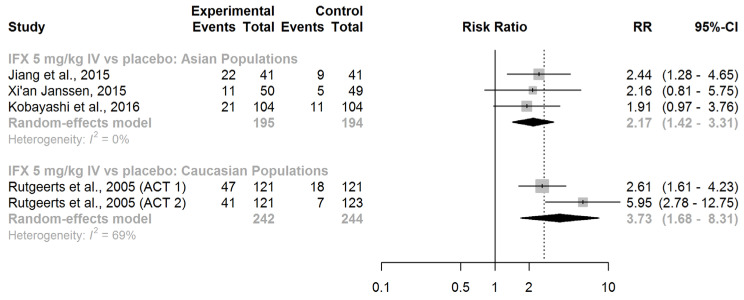
Induction of clinical remission. Abbreviations: IFX, infliximab; IV, intravenous; RR, risk ratio; CI, confidence interval, [40,41,42,43].

In adults with moderately to severely active UC who were treatment-naive with biologic therapies, IFX (given intravenously at a dose of 5 mg/kg at weeks 0, 2, and 6) displayed efficacy in inducing clinical remission, both in Asian (RR = 2.17, 95% CI: 1.42–3.31) and Caucasian patients (RR = 3.73, 95% CI: 1.68–8.31; Figure 3).

Upon comparing the race-specific pooled RRs, the difference was not significant (ratio of RRs = 0.58, 95% CI: 0.24–1.44; *z*-score = −1.17; *p*-value for interaction = 0.24). We found no substantial evidence of effect modification by race regarding the efficacy of IFX in inducing clinical remission in patients with active UC.

#### 3.2.3. Induction of Mucosal Healing

**Figure 4:**
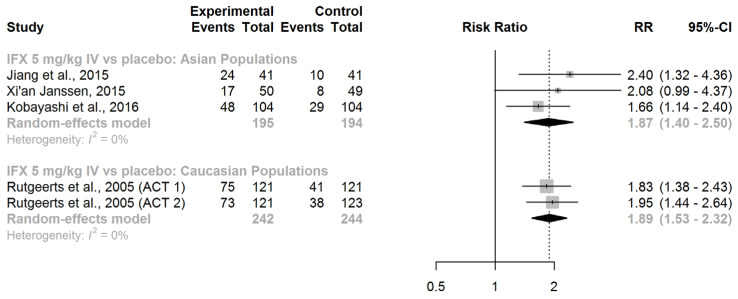
Induction of mucosal healing. Abbreviations: IFX, infliximab; IV, intravenous; RR, risk ratio; CI, confidence interval, [40,41,42,43].

Again, IFX (administered intravenously at a dose of 5 mg/kg at weeks 0, 2, and 6) exhibited notable efficacy in eliciting mucosal healing in UC patients who had not been previously exposed to biological treatments. This effect was similar in Asian (RR = 1.87, 95% CI: 1.40–2.50) and Caucasian populations (RR = 1.89, 95% CI: 1.53–2.32), as shown in Figure 4.

A comparison of the race-specific pooled effect estimates did not show any difference (ratio of RRs = 0.99, 95% CI: 0.69–1.41; *z*-score = −0.06; *p*-value for interaction = 0.95). Thus, we found no evidence of effect modification by race concerning the efficacy of IFX in inducing mucosal healing in patients with active UC.

#### 3.2.4. Maintenance of Clinical Remission

**Figure 5:**
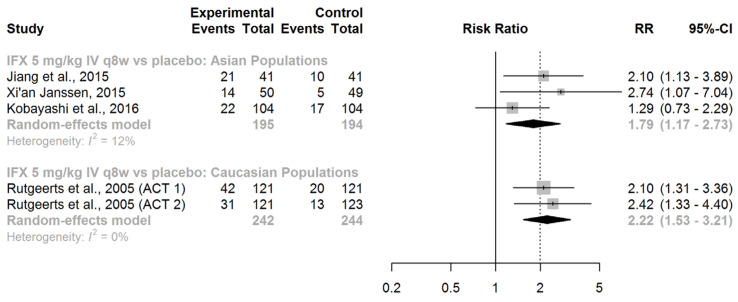
Maintenance of clinical remission. Abbreviations: IFX, infliximab; IV, intravenous; RR, risk ratio; CI, confidence interval; q8w, every 8 weeks, [40,41,42,43].

IFX (given intravenously at a dose of 5 mg/kg every 8 weeks) showed significant superiority over placebo in maintaining clinical remission in both Asian (RR = 1.79, 95% CI: 1.17–2.73) and Caucasian patients (RR = 2.22, 95% CI: 1.53–3.21; Figure 5).

When we compared the race-specific pooled RRs, no significant difference was noted (ratio of RRs = 0.81, 95% CI: 0.46–1.42; *z*-score = −0.75; *p*-value for interaction = 0.45). We found no evidence of effect modification by race regarding the efficacy of IFX in maintaining clinical remission in patients with UC.

#### 3.2.5. Maintenance of Mucosal Healing

**Figure 6:**
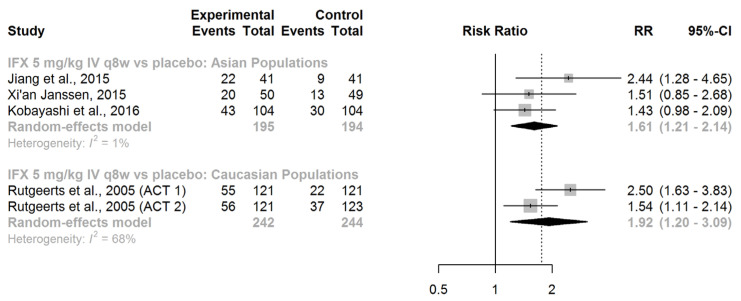
Maintenance of mucosal healing. Abbreviations: IFX, infliximab; IV, intravenous; RR, risk ratio; CI, confidence interval; q8w, every 8 weeks, [40,41,42,43].

IFX (given intravenously at a dose of 5 mg/kg every 8 weeks) demonstrated superiority over placebo in maintaining mucosal healing for both Asian patients (RR = 1.61, 95% CI: 1.21–2.14) and Caucasian patients (RR = 1.92, 95% CI: 1.20–3.09; Figure 6).

A comparison of the race-specific summary effect estimates did not reveal any significant difference (ratio of RRs = 0.84, 95% CI: 0.48–1.46; *z*-score = −0.63; *p*-value for interaction = 0.53). We found no evidence of effect modification by race concerning the efficacy of IFX in maintaining mucosal healing in patients with UC.

## 4. Discussion

In recent years, targeted immune modulators have considerably improved the care of patients with UC; nonetheless, uncertainties persist regarding the optimal selection of these drugs to maximize effectiveness, as well as whether racial disparities exist that could affect treatment outcomes and guide therapeutic choices. In our analysis of high-quality aggregate data from randomized, double-blind, placebo-controlled studies, no substantial evidence of racial differences was found regarding the efficacy of IFX in inducing clinical response, clinical remission, and mucosal healing, or maintaining clinical remission and mucosal healing, between Asian and Caucasian populations with moderate-to-severe UC. To the best of our knowledge, this study is the first to specifically examine whether race modifies the treatment efficacy of IFX in UC.

There is a paucity of research on the interaction between race and the effectiveness of advanced therapies in UC. In the first study conducted in this field [30], Greywoode and colleagues investigated racial differences in the efficacy of golimumab in UC trials [44,45] and concluded that individuals from racial minority groups (Asian, Black, or other races) exhibited a lower likelihood of achieving clinical response, clinical remission, and endoscopic healing compared to White patients. However, the authors neither presented race-specific estimates of the drug efficacy nor provided a ratio of the race-specific estimates for each outcome, nor did they report *p*-values from tests of interaction. Furthermore, contrary to their results, the efficacy of golimumab in a clinical trial of Japanese patients [46] was shown to be similar to that observed in golimumab trials predominantly involving White participants [44,45].

At this point, the fundamental question is whether one should expect racial differences in drug response, and whether such differences would be reliable for guiding therapeutic decisions. A debate has persisted for several years regarding the use of “race” in clinical research and practice. Some authors have suggested that race holds little or no biological significance and, therefore, should have limited importance in treatment decisions [47,48], while others have insisted that a patient’s race can and should influence physicians’ therapeutic decisions [49]. Current evidence, however, suggests that race is a poor proxy for drug response: Race disappears when examining the human genome. What people consider as racial differences comprise only 0.01% of our genes; geneticists agree that most of the genetic variation occurs within races, not between them [50].

In fact, race lacks a biological definition and has a fluid, political, and social meaning that is independent of science [51]. Accordingly, the American Medical Association has declared that race is a social construct rather than a biological one, recognizing *“race as a socially constructed category different from ethnicity, genetic ancestry, or biology”* and aiming to *“end the misinterpretation of race as a biological category defined by genetic traits or biological differences”* [52]. Hence, when research overly emphasizes race, it fails to recognize the social and environmental determinants of health that are the true drivers of racial disparities in health outcomes [53].

Overall, our study findings do not support the hypothesis that race modifies the treatment efficacy of IFX in UC. Yet, our analysis has limitations that merit consideration: First, our assessment of effect modification by race was based on between-study comparisons, which might introduce bias if unmeasured covariates among studies influence relative treatment effects [54]. Nevertheless, all studies included in this analysis exhibited homogeneity in terms of patient characteristics (i.e., adults with moderate to severe UC (defined with a Mayo score of 6–12 points, with endoscopic subscore of 2 or 3) who had not been previously exposed to biologics) and study methods (i.e., randomized, double-blind placebo-controlled design; uniform protocolized IFX treatment across trials; and the use of the Mayo score to define and measure study outcomes). Second, we were unable to assess treatment efficacy for racial groups other than Asian and Caucasian due to the minimal representation of non-White participants in the ACT trials [43], comprising only about 5% of the total, and the lack of reported race-specific efficacy results. Therefore, our findings may not be readily generalizable to Black and other racial groups.

Nonetheless, several strengths support the reliability and robustness of our analysis: First, we synthesized data from studies judged to be at low RoB in all key domains, except for the observed decrease in complete follow-up rates during maintenance phases. Second, we employed random-effect models to synthesize evidence within race-specific groups, thus allowing true effects to differ among studies [54,55]. Third, we used appropriate statistical methods to assess racial differences in the efficacy of IFX treatment between Asian and Caucasian populations. However, it is essential to interpret the results of the current analysis with caution when making clinical and health policy decisions, as “the absence of evidence is not evidence of absence” [56] of an interaction between treatment and race, especially because the statistical power of subgroup analyses in evidence syntheses is rather limited [57].

In conclusion, our study challenges previous assumptions about racial disparities in IFX efficacy for UC through the analysis of high-quality data from randomized trials. This approach provides valuable insights into the interplay of race and treatment outcomes. Our work not only advances scientific understanding, but also underscores the importance of more inclusive research practices. Ensuring the efficacy of advanced therapies for a broad spectrum of patients necessitates research that encompasses diverse populations. However, clinical trials have been criticized for insufficient representation of various racial minorities [58,59,60]. Future research should prioritize expanding the inclusion of racial groups, such as Blacks and Asians, in UC studies. This is crucial for a comprehensive evaluation of racial disparities in the effectiveness of advanced therapies, including but not limited to IFX, in the context of treating UC.

## Figures and Tables

**Table 1:** Randomized, placebo-controlled trials assessing IFX therapy in ulcerative colitis.

Study; Population Race	Acronym; ClinicalTrials.gov ID	Study Groups, Intervention Parameters, Number of Patients	Follow-Up
Jiang et al., 2015 [40]; Chinese, 100% of patients		IFX (5 mg/kg IV at wks 0, 2, 6, and q8w thereafter) (*n* = 41) vs. PBO (*n* = 41)	30 wks
Xi’an Janssen, 2015 [41]; Chinese, 100% of patients	NCT01551290	IFX (5 mg/kg IV at wks 0, 2, 6, and q8w thereafter) (*n* = 50) vs. PBO (*n* = 49)	26 wks
Kobayashi et al., 2016 [42]; Japanese, 100% of patients	Japic CTI-060298	IFX (5 mg/kg IV at wks 0, 2, 6, and q8w thereafter) (*n* = 104) vs. PBO (*n* = 104)	30 wks
Rutgeerts et al., 2005 [43]; Caucasians, 94% of patients	ACT 1; NCT00036439	IFX (5 mg/kg IV at wks 0, 2, 6, and q8w thereafter) (*n* = 121) vs. PBO (*n* = 121)	54 wks
Rutgeerts et al., 2005 [43]; Caucasians, 95% of patients	ACT 2; NCT00096655	IFX (5 mg/kg IV at wks 0, 2, 6, and q8w thereafter) (*n* = 121) vs. PBO (*n* = 123)	30 wks

Abbreviations: IFX, infliximab; IV, intravenous; wks, weeks; q8w, every 8 weeks; PBO, placebo. Footnote: We considered only data for dosage and administration as specified in the SPC.

## Data Availability

All relevant data are included in the published article.
